# A systematic review of integrated working between care homes and health care services

**DOI:** 10.1186/1472-6963-11-320

**Published:** 2011-11-24

**Authors:** Sue L Davies, Claire Goodman, Frances Bunn, Christina Victor, Angela Dickinson, Steve Iliffe, Heather Gage, Wendy Martin, Katherine Froggatt

**Affiliations:** 1 Centre for Research in Primary and Community Care, University of Hertfordshire, Hatfield, AL10 9AB, UK; 2 School of Health Sciences and Social Care, Brunel University, Middlesex UB8 3PH, UK; 3 Department of Primary Care and Population Sciences, University College London, NW3 2PF, UK; 4 Department of Economics, University of Surrey, GU2 7XH, UK; 5 Institute of Health Research, Lancaster University, LA1 4YT, UK

## Abstract

**Background:**

In the UK there are almost three times as many beds in care homes as in National Health Service (NHS) hospitals. Care homes rely on primary health care for access to medical care and specialist services. Repeated policy documents and government reviews register concern about how health care works with independent providers, and the need to increase the equity, continuity and quality of medical care for care homes. Despite multiple initiatives, it is not known if some approaches to service delivery are more effective in promoting integrated working between the NHS and care homes. This study aims to evaluate the different integrated approaches to health care services supporting older people in care homes, and identify barriers and facilitators to integrated working.

**Methods:**

A systematic review was conducted using Medline (PubMed), CINAHL, BNI, EMBASE, PsycInfo, DH Data, Kings Fund, Web of Science (WoS incl. SCI, SSCI, HCI) and the Cochrane Library incl. DARE. Studies were included if they evaluated the effectiveness of integrated working between primary health care professionals and care homes, or identified barriers and facilitators to integrated working. Studies were quality assessed; data was extracted on health, service use, cost and process related outcomes. A modified narrative synthesis approach was used to compare and contrast integration using the principles of framework analysis.

**Results:**

Seventeen studies were included; 10 quantitative studies, two process evaluations, one mixed methods study and four qualitative. The majority were carried out in nursing homes. They were characterised by heterogeneity of topic, interventions, methodology and outcomes. Most quantitative studies reported limited effects of the intervention; there was insufficient information to evaluate cost. Facilitators to integrated working included care home managers' support and protected time for staff training. Studies with the potential for integrated working were longer in duration.

**Conclusions:**

Despite evidence about what inhibits and facilitates integrated working there was limited evidence about what the outcomes of different approaches to integrated care between health service and care homes might be. The majority of studies only achieved integrated working at the patient level of care and the focus on health service defined problems and outcome measures did not incorporate the priorities of residents or acknowledge the skills of care home staff. There is a need for more research to understand how integrated working is achieved and to test the effect of different approaches on cost, staff satisfaction and resident outcomes.

## Background

In the UK care homes are the major provider of long term and intermediate care for older people [1-3]. There are 18, 255 care homes providing 459, 448 beds, almost three times as many as the 167, 000 hospital beds available [4]. Although people living in care homes have complex needs and represent the oldest and most frail of the older population in the UK, research consistently demonstrates that they have erratic access to NHS services, particularly those that offer specialist expertise in areas such as dementia and end of life care [5-9].

Inappropriate and unplanned hospital admissions, recognition of unmet health needs, concerns about supporting patient dignity, end of life care and access to health services have triggered multiple care home specific policy initiatives and interventions [10,11]. A consultation event that involved care home and health care representatives identified multiple examples of the NHS working with care homes to improve information exchange, palliative care, reduce falls, and unplanned admissions to hospital [12]. These interventions often involve the introduction of specialist health workers and teams or problem specific workers to achieve the desired outcomes [13,14].

Primary health care services in England spend significant amounts of time providing care for older people resident in these settings [15,16,7,8] (Goodman, C et al: Can clinical benchmarking improve bowel care in care homes for older people? Final report submitted to the DoH Nursing Quality Research Initiative PRP, Centre for Research in Primary and Community Care, University of Hertfordshire, 2007). However, relatively little is known about how health care services work with the (largely unqualified) workforce to provide care to a population that has complex physical and medication needs, experiences high level of cognitive impairment, depression and is in the last few years of life [17,18]. The involvement of health care services in care home settings is often defined by what care home staff are not allowed to do rather than a clear understanding of how the two sectors complement each other, or work together [19]. In addition, it cannot be assumed that health service definitions of problems and services reflect how older people and care home staff define health needs and the types of health care they would like (Evans, C: The analysis of experiences and representations of older people's health in care homes to develop primary care nursing practice, unpublished PhD King's College London, 2008).

Initiatives that support continuity and integration of care for older people with complex needs across health and social care with public and private providers are increasingly recognised as important for continuity and quality of care [20,21]. Integration of service provision can be defined as 'a single system of needs assessment, commissioning and/or service provision that aims to promote alignment and collaboration between the cure and care sectors [22]. There are different levels of integration between health care services [23]. In the context of integrated working with care homes, these can be summarised as:

### Patient/Micro level

Close collaboration between different health care professionals and care home staff e.g for the benefit of individual patients.

### Organisational/Meso level

Organisational or clinical structures and processes designed to enable teams and/or organisations to work collaboratively towards common goals (e.g. integrated health and social care teams).

### Strategic/Macro level

Integration of structures and processes that link organisations and support shared strategic planning and development for example, when health care services jointly fund initiatives in care homes [24,25].

To understand the evidence for the benefits of different approaches to health care services supporting older people in care homes, we conducted a systematic review to identify studies using integrated working between primary health care professionals and care homes for older people; evaluated their impact on the health and well being of older people in care homes, and identified barriers and facilitators to integrated working.

## Methods

The review was conducted according to inclusion criteria and methods pre-specified in a protocol developed by the authors before the review began.

### Inclusion criteria

We included interventions designed to develop, promote or facilitate integrated working between care home or nursing home staff and health care practitioners. Interventions that involved staff going in to provide education or training to care home/nursing home staff were included as long as there was some description of joint working or collaboration. We excluded studies where staff were employed specifically for the purpose of the research without consideration of how the findings might be integrated into ongoing practice (i.e. project staff introduced for a limited time to deliver a specific intervention). For a study to be included there had to be evidence of at least one of the following:

Clear evidence of joint working

Joint goals or care planning

Joint arrangements covering operational and strategic issues

Shared or single management arrangements

Joint commissioning at macro and micro levels

Studies also had to report at least one of the following outcomes:

Health and well being of older people (e.g. changes in health status, quality of life)

Service use (e.g. number of GP visits, hospital admissions)

Cost such as savings due to avoided hospitalisations

Process related outcomes (such as changes in quality of care, increased staff knowledge, uptake of training and education and professional satisfaction)

As the literature in this area is limited we included all studies that involved an element of evaluation. This included controlled and uncontrolled studies. However, because they are more susceptible to bias, studies without a control were used to describe and catalogue interventions rather than evaluate effectiveness. Process evaluations and qualitative studies including those using action research methodologies were included in order to identify facilitators and barriers to integrated working.

### Identification of studies

The electronic search strategy was conducted in February 2009. We searched the following electronic databases: Medline (PubMed), CINAHL, BNI, EMBASE, PsycInfo, DH Data, Kings Fund, Web of Science (WoS incl. SCI, SSCI, HCI) and the Cochrane Library incl. DARE. In addition, we contacted care home related interest groups and used lateral search techniques, such as checking reference lists of relevant papers, and using the 'Cited by' option on WoS, Google Scholar and Scopus, and the 'Related articles' option on PubMed and WoS. We applied no restrictions by date or country but included English language papers only. Details of the search terms used can be seen in Table 1.

**Table 1:** Search terms on PubMed (search terms were suitably adapted for other databases)

Component 1
Search "Delivery of Health Care, Integrated"[Mesh] OR integrated[ti] OR team[ti] OR interdisciplinary[ti] OR integration[ti] OR integral[ti] OR integrat*[ti] OR seamless[ti] OR continuity[ti] OR interface[ti] OR multidisciplinary[ti] OR multiprofessional[ti] OR multiagency[ti] OR interprofessional [ti] OR multi sector[ti] OR model*[ti] OR coordinat*[ti] OR partnership*[ti] OR tufh OR continu*[ti] OR interagenc*[ti] OR stakeholder*[ti] OR network*[ti] OR systems[ti] OR team*[ti] OR shared[ti] OR joined-up[ti] OR pooling[ti] OR vertical*[ti] OR horizontal*[ti] OR collaborat*[ti] OR cross organi*[ti] OR multi-professional[ti] or intermediate care[ti] or multi agency[ti] or multiagency[ti] OR managed care[ti] OR joint care[ti] OR ((individual[ti] or separate[ti]) AND budget) OR partner*[ti] OR all-inclusive[ti] OR in-reach[ti] OR chain[ti] OR comprehensive[ti] or total care[ti] OR interface[ti] OR "service interaction" OR seamless[ti] OR interagency[ti] OR "Patient Care Team"[MAJR]
AND
Search Family Physicians OR general pract*[ti] OR general physician*[ti] OR family doctor*[ti] OR general medicine[ti] OR Primary Health Care OR Continuity of Patient Care OR "primary care" OR continuity of care OR physician*[ti] OR "Physicians"[Majr:NoExp] OR "Physicians, Family"[Majr] OR "Physician Assistants"[MeSH Terms] OR"Nurse Practitioners"[MeSH Terms] OR "Physician's Practice Patterns"[MAJR] OR physician*[ti] or practitioner*[ti]
AND
Search Nursing Homes OR nursing home*[ti] OR "nursing home*" OR long-term care[ti] OR long term care [ti] OR nursing facilit*[ti] OR residential[ti] OR institutional care[ti] OR resident*[ti] OR continuing [ti] OR respite care OR nightingale home OR nightingale homes OR care home*[ti] OR long-term[ti] OR longterm[ti]
AND
Search geriatrics OR elderly OR older OR middle age OR middle-age OR senior OR frail OR care of elderly OR geriatric nursing OR geriatric assessment OR "Aged"[Mesh] OR "Health Services for the Aged"[Mesh] OR "Middle Aged"[Mesh] OR "Homes for the Aged"[Mesh] OR "Aged, 80 and over"[Mesh] OR senior*[ti] or pensioner*[ti] OR retire*[ti]
**Component 2: Simplified, focused searches involving two aspects of the subject:** **NHS/Primary Care/Nursing homes**
Search ("Physicians"[Majr:NoExp] OR "Physicians, Family"[Majr] OR "Physician Assistants"[MeSH Terms] OR"Nurse Practitioners"[MeSH Terms] OR "Physician's Practice Patterns"[MAJR] OR physician*[ti] OR practitioner*[ti] OR specialist*[ti] OR primary care[ti]) (nursing home*[ti] OR residential care[ti] OR care home*[ti] OR residential home*[ti])
**Nursing homes/Integrated Care**
Search (nursing home*[ti OR residential care[ti] OR care home*[ti] OR residential home*[ti]) (integrat*[ti] or team*[ti] or cooperation[ti] OR multidisciplinary[ti])
**Elderly/Integrated Care**
Search (elderly[ti] or older[ti] or geriatric*[ti] OR senior[ti]) (integrat*[ti] OR team*[ti]) AND (community OR nursing homes)

### Data extraction and synthesis

Electronic search results were downloaded into EndNote bibliographic software. Two reviewers independently (SD, FB) screened all titles and abstracts of citations identified by the electronic search, applied the selection criteria to potentially relevant papers, and extracted data from included studies using a standardised form. Any disagreements concerning studies to be included were resolved by consensus or by discussion with a third reviewer (CG).

Due to substantial heterogeneity in study design, interventions, participants and outcomes we did not pool studies in a meta-analysis. Instead a narrative summary of findings is presented and where possible we have reported dichotomous outcomes as relative risks (RR) and continuous data as mean differences (MD) (with 95% confidence intervals). Data in the evidence tables is presented with an indication of whether the intervention had a positive effect (+), a negative effect (-), or no statistically significant effect (0). The qualitative studies were used to generate a list of potential barriers and facilitators to integrated working. Each paper was systematically read by two researchers (SD, CV) to highlight any factors that may have impacted on the process, both those that were explicitly referred to by the authors and those identified by the reviewers within the papers' narratives.

The quality of the included studies was assessed using design assessment checklists informed by the Cochrane Collaboration risk of bias tool [26] and Spencer et al's quality assessment checklist for qualitative studies [27]. The core quality-assessment domains are summarised in Table 2. As other non controlled studies were used to inform contextual understanding rather than evaluate effectiveness they were not formally quality assessed.

**Table 2:** Quality assessment criteria by study type

Randomised controlled trials all scored as *Yes/No/Unclear*	
*Sequence generation *	Was the allocation sequence adequately generated?
*Allocation concealment *	Was allocation adequately concealed?
*Blinding *	Was knowledge of the allocation intervention adequately concealed from outcome assessors?
*Incomplete outcome data*-	Was this adequately addressed for each outcome?
*Selective outcome reporting *	Are reports of the study free of suggestion of selective outcome reporting?

**Controlled studies (without randomisation) all scored as *Yes/No/Unclear***	

*Baseline results reported*	Were baseline results reported for each group?
*Groups balanced at baseline*	Were there any significant differences in the groups at baseline?
*Blinding *	Was knowledge of the allocation intervention adequately concealed from outcome assessors?
*Incomplete outcome data*-	Was this adequately addressed for each outcome?
*Selective outcome reporting *	Are reports of the study free of suggestion of selective outcome reporting?

**Qualitative studies - *Scored as fully or mostly, partly or not at all***	

*Scope and purpose*	*e.g*. clearly stated question, clear outline of theoretical framework
*Design*	*e.g*. discussion of why particular approach/methods chosen
*Sample*	*e.g*. adequate description of sample used and how sample identified and recruited
*Data collection*	*e.g*. systematic documentation of tools/guides/researcher role, recording methods explicit
*Analysis*	*e.g*. documentation of analytic tools/methods used, evidence of rigorous/systematic analysis
*Reliability and validity*	*e.g*. presentation of original data, how categories/concepts/themes developed and were they checked by more than one author, interpretation, how theories developed
*Generalisability*	*e.g*. sufficient evidence for generalisability or limits made clear by author
*Credibility/plausibility*	*e.g*. provides evidence that resonates with other knowledge, results/conclusions supported by evidence

Data were extracted from each study on methodology, type of intervention, outcomes, participants, and location. In addition, an interpretive approach based on Kodner and Spreeuwenberg's (2002) work on integrated working, was used to compare and contrast the nature and level of integration across the studies using the principles of framework analysis [28]. Each study was categorised in terms of the degree of integration and the complexity classified as micro, meso and or macro. In addition, based on the assumption that care homes with a higher level of integration would show evidence of correspondingly greater levels of support and contact with health care professionals, each study was analysed to identify the amount of contact, support and training given by the health professionals involved in the study.

## Results

**Figure 1:**
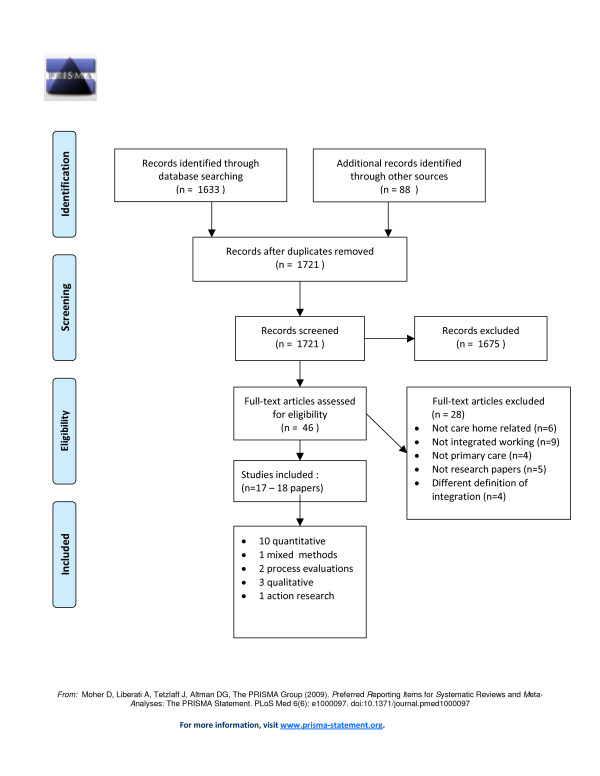
**PRISMA Flow Diagram**. Systematic review process from electronic searching to study inclusion.

Figure 1 shows the flow of studies through the selection process. Seventeen studies (reported in 18 papers) met our inclusion criteria.

### Description of studies

Ten studies were quantitative, (four of which were RCTs), one used mixed methods, two were process evaluations, three were qualitative and one was action research (see Table 3).

**Table 3:** Studies included in the systematic review of integrated working between care homes and health care services:

First Author, Year Title Study design	Research Question/aims and objectives	Study population, setting and country of study	Sample size/number of participants: Include power calculation if available	Description of intervention/ Study design	Main outcome variable(s)/ Areas of focus for qualitative studies	Main findings/ Conclusions
1. King, 2001 **Multidisciplinary case conference reviews: improving outcomes for nursing home residents, carers and health professionals** *Controlled study*	To determine whether multidisciplinary case conference reviews improved outcomes for nursing home residents and its impact on care staff.	**Population:** Older people in nursing homes **Setting:** 3 nursing homes **Country: Australia**	245 older people But only 75 residents were reviewed	Weekly case conference reviews, one review per resident, over 8 months attended by GPs, clinical pharmacist, senior nursing staff and other health professionals. Multidisciplinary discussion of all aspects of a resident's care to make recommendations and devise a management plan for the resident. Reviews were led by GPs with data collection by the pharmacist. Baseline and endpoint comparisons were made between residents who were reviewed and those who were not.	Resident outcomes included: medication use, administered medications and weekly cost, health status and quality of life. Carer outcomes were based on resident interaction, workload or personal/professional satisfaction.	• There were no significant reductions in medications orders, cost and mortality. 40% of the recommendations benefited residents, measured through their health status and quality of life. 26% of the recommendations benefited care staff, but no details were given. Multidisciplinary case conferences were seen as beneficial to patients and carers. Their future use was recommended.

2. Llewellyn-Jones, 1999 **Multifaceted shared care intervention for late life depression in residential care: randomised controlled trial**. *RCT*	To evaluate the effectiveness of a population based multifaceted shared care intervention for late life depression in residential care.	**Population:** Older people 65 years + with depression and no or low cognitive impairment **Setting:** Residential facility living in self care units and hostels **not **nursing homes (equivalent to residential care in UK) Residents were stratified and randomised to intervention or control **Country: Australia**	220 older people No power calculation	The shared care intervention included: 1. Multidisciplinary consultation and collaboration 2. Training of gps and carers in detection and management of depression 3. Depression related health education and activity programmes for residents. The control group received routine care.	Geriatric Depression Scale	There was a significant reduction in adjusted depression scores for residents in the intervention group. Multidisciplinary collaboration, staff education, health education and activity programmes can improve depression in older people in residential care.

3. Opie, 2002 **Challenging behaviours in nursing home residents with dementia: a randomised controlled trial of multidisciplinary interventions**. *RCT*	To test whether individually tailored psychosocial, nursing and medical interventions to nursing home residents with dementia will reduce the frequency and severity of behavioural symptoms.	**Population;** Nursing home residents with severe dementia rated by staff as having frequent, severe behavioural disturbances. **Setting: **42 Nursing homes **Country: Australia**	102 older people entered the study, (99 completed the 4 week trial, 2 RIPs 1 hospitalisation)	Residents selected on basis of CMAI scores and assigned to early or late intervention groups. Consultancy team with training in psychiatry, psychology and nursing met weekly for 30 minutes, to discuss referrals and formulate individualised care plans which were presented to nursing home staff to implement. Plans were reviewed at one week. 3 categories: medical, based on medication review, nursing, based on ADLs, and psychosocial including environment, sensory stimulation. The control was normal care, residents acted as their own controls by being in the early or late intervention groups.	Frequency and severity of disruptive behaviours and assessment of change by senior nursing staff. Tools included: Cohen-Mansfield Agitation Inventory (CMAI) which assesses frequency of 30 behaviours over previous 14 days Behaviour Assessment Graphical System (BAGS) which records a combined frequency and disruption score every hour for 24 hours.	There was a slight reduction in the daily observed counts of challenging behaviours. Individualised, multidisciplinary interventions appear to reduce the frequency and severity of challenging behaviours in nursing homes

4. Schmidt, 1998 **The Impact of Regular Multidisciplinary Team Interventions on Psychotropic Prescribing in Swedish Nursing Homes** *RCT*	To evaluate the impact of regular multidisciplinary team interventions on the quantity and quality of psychotropic drug prescribing in nursing homes Aim was to improve prescribing through better teamwork amongst physicians, pharmacists, nurses and nursing assistants	**Population:** Long term residents, 42% dementia, 5% psychotic disorder, 7% depression **Setting: **33 Nursing homes **Country: Sweden**	1854 residents In 15 experimental homes and 18 control homes	Regular multidisciplinary team meetings over 12 months to discuss individual residents drug use. Training was provided for pharmacists but not for other staff. Control homes provided normal care.	Baseline and 12 month post resident medications	After 12 months the intervention group showed an improvement in the prescribing of hypnotics only. Prescribing practices can be improved through better teamwork between health care and nursing home staff using clinical guidelines.

5. Vu, 2007 **Cost-effectiveness of multidisciplinary wound care in nursing homes: a pseudo-randomized pragmatic cluster trial** *Pseudo RCT*	Trial to test the hypothesis that trained pharmacists and nurses working in collaboration with a wound treatment protocol would improve the wound healing and save costs.	**Population:** 176 residents with leg or pressure wounds **Setting:** 44 high care nursing homes **Country: **Australia	Based on an assumed improvement in the healing rate from 15% to 30%, 108 wounds per arm were required to have an 80% chance of detecting a two-fold increase in healing rates at a significance level of 5%. To adjust for clustering this number was increased to 151 in each group.	Residents in the intervention arm received standardised treatment from a wound care team comprised of trained community pharmacists and nurses. A standard treatment protocol was developed based on the colour, depth and exudate method for assessing wounds and the group's clinical and academic experience. They met weekly to discuss any new wounds and treatment options within the protocol. Both nurses and pharmacists received training on wound healing and management.	Treatment recommendations, frequency and detail of dressing changes, measurement and photos of wounds, SF36, Assessment of Quality of Life index, Brief Pain Inventory - measures wound pain, total estimated cost of treatment per wound including, staff time, training, wound care products and waste disposal.	During the trial more wounds healed in the intervention than in the control group but this was not significant. The mean treatment cost of wound healing was significantly less in the intervention group. Standardised treatment by a multidisciplinary wound care team cut costs and improved chronic wound healing in nursing homes.

6. Crotty 2004 **An outreach geriatric medication advisory service in residential aged care: a randomised controlled trial of case conferencing**. *Cluster RCT*	Evaluate the impact of multidisciplinary case conferences on the appropriateness of medications and on patient behaviours in residential care	**Population: **residents with medication problems/challenging behaviours **Setting: **10 High-level aged care facilities **Country: Australia**	154 residents recruited with 54 in control, 50 in intervention, 50 in within facility control group 5 facilities randomised to the intervention and 5 to the control Staff nominated 20 residents for the intervention and 10 for the control, based on 2 criteria: Residents with a difficult behaviour they would like advice on, those prescribed 5+ medications An effect size based on patients aged 65 + with polypharmacy of 0.9 in the MAI between the intervention and control groups (power 0.9, type 1 error of 0.05) would be detected with 28 residents in each group	2 multidisciplinary case conferences chaired by the resident's GP, a geriatrician, pharmacist and residential care staff held at the nursing home for each resident. All facilities received a half day workshop on using the toolkit for challenging behaviour All residents had their medication chart reviewed pre and post intervention by an independent pharmacist using the MAI	Assessed at baseline and 3 months Primary outcome the Medication Appropriateness Index (MAI) Nursing Home Behaviour Problem Scale for each resident	There was a significant improvement in appropriate medication in the intervention group compared with the control group. Resident behaviours were unchanged after the intervention.

7. Joseph 1998 **Managed Primary Care of Nursing Home Residents** *Cohort study*	To measure the rates of hospital use and mortality of nursing home residents who received their primary care from practitioner-physician teams.	**Population: **older long term residents of nursing homes enrolled in Medicare HMO **Setting:** 30 nursing homes in Southern California **Country: USA**	307 nursing home residents	Primary care by accessible interdisciplinary team including physicians, nurse practitioners, and nursing home staff supported by clinical guidelines, continuous improvement techniques and increased availability of clinical services at the nursing homes.	Demographics, mortality, hospital days, minimum data sets	Integrated working between doctors, nurse practitioners and nursing home staff can reduce nursing home resident's hospital use.

8. Kane 2004 **Effect of an Innovative Medicare Managed Care Program on the Quality of Care for Nursing Home Residents** *Controlled study*	To assess the quality of care provided by Medicare HMO targeted specifically at nursing home residents, employing nurse practitioners to provide additional primary care to the physicians.	**Population: **Long stay nursing home residents **Setting: **Nursing homes **Country: USA**	44 Evercare homes 44 control homes 2 control groups a) other residents in same homes not enrolled in Evercare b) residents in homes in same geographical area that did not participate in Evercare	Evercare model of managed care using nurse practitioners to provide additional primary care over and above that provided by physicians.	4 aspects of quality: mortality, preventable hospitalisations, quality indicators, derived from the Minimum Data set and changes in functioning.	The Evercare mortality rate was significantly lower than the control-in group but not the control-out group. The Evercare residents had fewer preventable hospitalisation s the difference was significant for one of the control groups.

9. Goodman 2007 *Controlled study*	To assess whether clinical benchmarking can be incorporated into care homes for older people with the support of NHS primary care nursing staff	**Population** Older people in residential care homes **Setting: **7 residential care homes (6 +1 pilot home) **Country: UK**	46 Care home staff and 154 older people from 6 residential care homes 12 district nurses from 6 district nursing teams in 3 PCTs.	3 intervention care homes used Essence of Care benchmarking in relation to resident's bowel care, joint implementation for all residents by care home staff working together with senior district nursing staff over six months. Regular benchmarking meetings to discuss, plan and implement specific aspects of bowel related health promotion and continence care that would be suitable for residents. DN led bowel care training sessions for other care staff in the care homes. Non-intervention care homes received usual care from their district nursing teams	Main outcome variables were bowel related problems captured in a bowel diary recorded for residents pre and post intervention and related hospital admissions, medication and continence product use, time spent on bowel related activities, staff satisfaction and turnover.	Clinical benchmarking could be utilised in care homes as part of everyday working with district nurses and used few resources. However, commitment by both parties and mutual trust was necessary for the process to be successful. Bowel care was complex and challenging for care staff especially where older people were cognitively impaired. There was no significant reduction in bowel related problems but some evidence of improved documentation and appropriate prescribing.

10. Szczepura, 2008 **In-reach specialist nursing teams for residential care homes: uptake of services, impact on care provision and cost-effectiveness**. *Economic evaluation*	Evaluation of a dedicated nursing and physiotherapy in-reach team (IRT)	**Population: **older people in care homes **Setting**; 4 residential care homes **Country: UK**	131 residents	IRT gives 24 hour cover 7 days a week - a specialist team offers support and onsite care for up to 15 beds for specialist nursing care to prevent transfer to hospital or nursing home. It also supports care home staff through health training up to NVQ level 3.	Cost of the service Number of referrals to the service Reasons for referral/visits by team Hospitalisations and nursing home transers avoided	IRT resulted in savings through reduced hospitalisations, early discharges, delayed transfers to nursing homes and illness recognition. Introduction of an in-reach team was at least cost neutral. It also benefited the care home staff through training which enhanced the quality of care and reduced the transfer of residents to other care facilities.

11. Proctor, 1998 **An observational study to evaluate the impact of a specialist outreach team on the quality of care in nursing and residential homes** *Quantitative - non-participant observation*	To assess the applicability of a training and support programme for care staff in nursing and residential homes on the quality of staff-resident interaction	**Population:** Older people considered by staff to have problems in terms of behaviour, social functioning or psychiatric symptoms **Setting: **5 residential homes, 1 nursing home **Country: UK**	12 residents - 2 from each home 51 care home staff	1. Staff training over 6 months included Seminars provided by a multidisciplinary team including old age psychiatrists, nurses, doctors and OTs. A behavioural approach to care planning to help staff plan and implement care plans for individual residents. Training was given by a psychiatric nurse with weekly visits to staff	Resident behaviour and staff contact was recorded through non-participant observation prior to the training, 3 and 6 months post Activities recorded were based on QUIS - Quality of Interactions Schedule (Dean et al, 1993)	There was a significant increase in the proportion of time that staff spent in positive interactions with residents (direct care p < 0.002, social contact p < 0.05) and levels of resident activity increased (p < 0.001).

12. Knight, 2007 **All-Wales integrated care pathway project for care homes** *Process evaluation/audit*	To facilitate the implementation of ICP into care homes through negotiation with local palliative care providers to improve the care for dying patients	**Population:** Older people in nursing homes **Setting:** 29 nursing homes in Wales **Country: **UK	130 older people pre-intervention, 133 post intervention	Introduction of an integrated care pathway for dying patients in care homes. Other support: • Education subgroup • ICP education pack • Teaching sessions • Syringe driver training • Matron forums • Informal training/support	Pre and post ICP audit of dying patient's notes to measure their quality. Pre-audit highlighted poor communication, symptom control, and lack of staff end of life care education.	The re-audit indicated an improvement in recording end of life care. ICP use in the care homes had increased from 3 to 31% in one year. Recording of events and documentation remained poor.

13. Mathews, 2006 **Using the Liverpool Care Pathway in a nursing home** *Process evaluation/* *Audit*	Aim to illustrate how collaborative working in a nursing home using the Liverpool Care Pathway(LCP) can enhance end of life patient care and improve palliative care education	**Population:** Older people resident in a nursing home **Setting: **1 nursing home **Country: UK**	150 residents with 50 bed contracted out to the NHS for end of life care	Pilot study to introduce LCP into a nursing home. LCP discussed with GPs, pharmacist and ambulance service. Trained nursing staff received 3 hours of palliative care training including using LCP. Followed by implementation of the LCP for patients.	Focus on improving documentation and symptom control of patients	An audit of the first 10 patients on the LCP showed an improvement in documentation and assessment of symptoms. Staff felt that the training should be extended to health care assistants. A steering group was also set up to discuss the pathway and training needs.

14. Doherty, 2008 **Examining the impact of a specialist care homes support team** *Qualitative*	To examine the work the work and perceived impact of a dedicated care homes support team Aim of the care homes support team was to enable staff to manage the health and social care needs of residents to avoid unnecessary admission to hospital	**Population:** Older people in care homes **Setting:** 29 Care homes? residential **Country: UK**	19 care home managers, 13 CHST including specialist older peoples nurse, pharmacist, GP, and Senior managers in PCT interviewed 32+ participants interviewed	Intensive component:: 5 care homes CHST promoted practice development through action plans focusing on staff identified needs Extensive component: 29 homes where CHST acted as a resource in terms of information sharing and networking but no development working	Processes, working methods and outcomes of the care home support team	Statistical analysis did not support the effectiveness of the care homes support team, but the qualitative data showed the impact of the team through empowering staff, increased quality of life and access to services for residents and professional development for staff.

15. Hasson, 2008 **The palliative care link nurse role in nursing homes: barriers and facilitators** *Qualitative*	To explore link nurses' views and experiences regarding the development, barriers and facilitators to the implementation of the role in palliative care in the nursing home	**Population: **Older people in nursing homes **Setting: **33 nursing homes **Country: UK**	33 nursing homes 14 link nurses in 3 focus groups	Link nurse initiative - 3 phases over 3 years: 1. Training needs or nurses and nursing assistants assessed 2. Palliative care educational programme for staff and identification of link nurses identified in nursing homes 3. Evaluation of link nurses by nursing home staff	Topics in focus groups included; link nurse preparation, barriers and facilitators to delivery of education in the home	The link nurse system had the potential to improve palliative care in nursing homes. Facilitators included external and peer support, monthly meetings and access to information. Barriers included the transient workforce and a lack of preparation for the role.

16. Avis 1999 **Evaluation of a project providing community palliative care support to nursing homes** *Qualitative*	Evaluation of project to extend 'hospice standards' of palliative care to nursing homes	**Population:** 231 Nursing home residents **Setting: **Nursing homes with registered palliative care beds **Country: UK**	2 Questionnaire surveys of 39 & 43 matrons of nursing homes, at 6 months and at the end of the project 35 Interviews with local stakeholders	Project was implemented by a nurse advisor and a peer support group of 6 district nurses who delivered the service to nursing homes. Nursing home staff made referrals to the team who responded by visiting and assisting in assessments and care plans for residents. 1^st ^phase involved assessment of services required by nursing homes identified by matrons. Focus on 3 areas: advice on individual care problems, training and support on palliative care, pain, symptom control, accessing specialist advice and offering support to relatives and residents including bereavement counselling.	Interviews explored participant's understanding of the project, their perceptions of issued involved in providing palliative care, benefits, limitations for staff and residents. Questionnaires were used to rate project performance, access, response time, liaison, benefits and limitations of the project. Services were also rated in order of their importance for care homes and residents.	The project helped to overcome the barriers to care between NHS services and the independent sector. Care home isolation was decreased through assistance with individual care and better access to specialist advice and training.

17. Hockley 2005 (primary) **Promoting end of life care in nursing homes using an integrated care pathway for the last days of life** 18. Watson 2006 (secondary) **Barriers to implementing an integrated care pathway for the last days of life in nursing homes** *Action research*	To promote quality end of life care in nursing homes using an integrated care pathway document. Explores the barriers that needed to be overcome during the implementation of an integrated care pathway for eol care	**Population:** Older people in nursing homes **Setting: **8 independent nursing homes **Country: UK**		Use of action research to promote collaboration between staff in nursing homes and the research team, empower staff in practice of eol care and promote sustainable eol care once study complete. - Core research team of 3 nurses with palliative care and action research experience, + 2 champions were identified in each care home Facilitation to implement ICP: - Monthly action learning sets for champions, monthly collaborative learning groups for all staff to reflect on eol care and ICP documents of residents who had died, clinical support from nurse specialist researcher.	Interviews to explore the respondents' understanding of the project, their perceptions of the issues in providing palliative nursing care and the benefits and limitations of the project for staff and residents Questionnaires focussed on: their use of the project, access, response time and liaison, perceptions of the benefits and limitations and the difficulties experienced in providing palliative. Data was also collected through field notes, action learning sets, monthly collaborative learning groups.	Dying became more central to nursing home work. Five main themes emerged, a greater openness to death, recognition of dying, better teamwork, using palliative care knowledge to influence practice and better communication.

Nine were conducted in the UK, five in Australia, two in the USA and one in Sweden. Eleven (65%) studies were conducted in nursing homes, five in residential homes and one in a combination of both. Study participants included residents, relatives, care home staff both residential and nursing, and health professionals including general practitioners, district nurses, nurse specialists, pharmacists, psychiatrists and psychologists.

Seven studies were focused on individual care, for example, specific health care needs such as end of life [29-33] or wound care [34] and dementia [35]. Six studies focused on residents' needs as a group, such as detection and treatment of depression [36], bowel related problems (Goodman, C. et al: Can clinical benchmarking improve bowel care in care homes for older people? Final report submitted to the DoH Nursing Quality Research Initiative PRP, Centre for Research in Primary and Community Care, University of Hertfordshire, 2007.) and or supporting the care home staff interactions with residents through training [37] and improved prescribing [38-40]. A further four papers were service evaluations such as an in-reach team for care homes [41], a care home support team [42], and nurse practitioners [43,44]. End of life care accounted for five papers [29-33], three of which focused on care pathways [30-32].

### Risk of bias

There were seven controlled studies of which four were RCTs. Although the RCTs could be expected to be less susceptible to bias than the non randomised studies the potential for bias in both groups of studies appeared to be high (see Tables 4 and 5).

**Table 4:** RCTs Quality assessment results

Study	Sequence generation adequate?	Allocation concealment adequate	Blinding of outcome assessment	Incomplete outcome data assessed?	Free from selective reporting?
Crotty 2004	Y	Y	N	Y	Y

Llewellyn-Jones 1999	Y	U	Y	N	Y

Opie 2002	Y	U	N	Y	Y

Schmidt 1998	U	U	U	U	Y

**Table 5:** Non randomised controlled studies quality assessment results

Study	Baseline results reported?	Groups balanced at baseline?	Blinding of outcome assessment	Incomplete outcome data assessed?	Free from selective reporting?
Goodman 2007	Y	Y	N	N	Y

King 2001	Y	N	N	Y	Y

Kane 2004	N	N	Y	N	Y

Vu 2007	Y	N	N	Y	Y

A number of the studies appeared underpowered and for many follow up was short. The qualitative studies employed a range of methodologies including action research, interviews, focus groups and questionnaires. As with the quantitative studies, the quality was low, only two out of four [30,33] had a clearly defined purpose and design. With one exception [33] descriptions of the study sample, data collection and analysis were inadequate and evidence of their credibility and transferability was limited (see Table 6).

**Table 6:** Quality review scores for qualitative papers.

*Study*	*Scope/purpose*	*Design*	*Sample*	*Data collection*	*Analysis*	*Reliability/validity*	*Generalisability/transferability*	*Credibility/integrity/plausibility*	*Ethics approval*
*Avis 1999*	~	-	-	-	-	-	-	~	-

*Doherty 2008*	~	+	~	-	~	-	-	+	+

*Hasson 2008*	+	+	+	+	+	+	~	+	+

*Hockley 2005*	+	+	~	~	-	~	~	+	+

Scoring key:

**+ **Fully or mostly scores 1

**- **Not at all

**~ **Partly scores 0.

### Effectiveness

The heterogeneity of outcomes and, in particular, the interventions meant that making comparisons between studies was problematic. Three studies looked at the effect on prescribing [38-40], three included mortality as an outcome [39,40,44] and two looked at disruptive behaviour [35,39]. The remaining outcomes, only included in single studies, were depression [36], hospital admissions [40], functional status [40], wound healing [34], and bowel related problems (Goodman, C et al: Can clinical benchmarking improve bowel care in care homes for older people? Final report submitted to the DoH Nursing Quality Research Initiative PRP, Centre for Research in Primary and Community Care, University of Hertfordshire, 2007). Full details of the results can be seen in Table 7. Although there were some improvements in outcomes, the majority of studies showed that the intervention had either mixed effects (that is improvement in one outcome but no effect or negative effect in another outcome), or no effect when compared with the control group. Insufficient information was available to evaluate the cost of integrated working between care homes and primary health care professionals.

**Table 7:** Results from RCTs and controlled studies

Study ID	Outcome	Main results at follow up (+) = positive effect, (-) = negative effect, (0) = no significant effect
Crotty 2004 RCT	Appropriate prescribing (medication appropriateness index)	Follow up at 3 months (NB - two control groups - one external and one within the facility (results presented for external control grp only))
		Change MAI score (+) Mean score (95% CI)Intervention 4.10 (2.11-6.10), Control 0.41 (-0.42-1.23), Difference p = 0.004
	Nursing home behaviour problem	Change NHBPS (0), Mean score (95% CI)Intervention 3.9 (-2.7-10.5), Control 1.2 (-9.1-11.6), P = 0.440
	Mortality	Mortality (0) No differences between groups (p = 0.304)

Goodman 2007 (non randomised controlled study)	Bowel related problems	Follow up at 6 months Normal bowel patterns (+) Intervention - significant increase in normal bowel patters, control grp - little change
	Medication and continence related product use	Prescription of laxatives (0) Increase in both groups but no statistically significant differences between groups p = 0.159
	Dependency (Barthel index)	Dependency (+) Mean change score p = 0.002 Intervention -0.02 (SD 3.1), Control -1.84 (SD 3.7)
	Bowel related hospital admission	1 admission in intervention grp, none in control (n = 120)

King 2001 (non randomised controlled study)		Follow up at 1 month. Data collected on 184 residents (75 reviewed, 109 not reviewed).
	Medication prescribed	Changes in medication prescribed - mean (SD) (0) Intervention -0.35 (2.56), Control -0.03 (1.90) P = 0.37
	Medication administered	Changes in medication administered - mean (SD) (0) Intervention -0.44 (2.45), Control 0.12 (1.84), P = 0.16
	Weekly Cost ($) - authors say study underpowered for this outcome	Weekly cost (0) Intervention -0.29 (10.80), Control 0.43 (12.16), P = 0.75
	Mortality (adjusted for length of time in home)	Mortality (0) Adjusted mortality data showed 6% of reviewed residents died compared to 15% of those not reviewed p = 0.07

Kane 2004 (controlled study) - evaluating EverCare		Follow up at 18 months 2 control groups a) other residents in same homes not enrolled in Evercare b) residents in homes in same geographical area that did not participate n Evercare Assessments at 6,12, 18 months (within 30 days)
	Mortality	Mortality Evercare rate significantly less than for control-in group but was slightly higher than control-out group (non significant)
	Preventable hospitalizations	Rates of preventable admissions lower in Evercare than for either control but only significant when compared to control-out. No differences in hospitalization rates overall. (0)
	Functional change	No significant differences in ADLs between Evercare and either control. (0)

Llewellyn-Jones 1999 RCT	Geriatric depression scale (score of ≥ 10 defined as depressed)	Follow up after 9.5 months Depression Unadjusted MD (0) -0.76 (-2.09, 0.57)
		Adjusted difference in change score (+) Multiple linear regression analysis Intervention group 1.87 improvement on scale compared to control group (95% CI 0.76, 2.97) p = 0.0011

Opie 2002 RCT (poor study design)	Frequency & severity of disruptive behaviours (Behaviour Assessment Graphical System and counts of certain behaviours)	Follow up at one month Frequency of disruptive behaviour (0) ANOVA revealed no statistically significant changes BAGS scores (0) No significant between group differences
	Assessment of change by senior nursing home staff - rated on 4 point scale(interviewed one month after completion of trial)	Assessment by staff No data reported on between group differences. Staff reported that the frequency of target behaviours had decreased in at least one behavioural category for 75% residents and that severity had decreased in at least one category for 60%.

Schmidt 1998 RCT	Proportion of pts with any psychotropic drug (from lists of residents prescriptions)	Follow up at 12 months Any psychotropic drug use (0) RR 0.97 (95% CI 0.92, 1.03)

Involves pharmacists	Proportion of residents with two or more drug classes (polymedicine)	Two or more drug classes (0) RR 1.02 (0.92, 1.13)
	Proportion of residents with therapeutic duplication (two or more drugs in same class)	Two or more drugs in same class RR 0.92 (0.76, 1.10)
	Number of drugs prescribed	Number of drugs prescribed (mean) 2.08% versus 2.20% Significant increase in average number of drugs prescribed in control before to after. No change in experimental homes.
	Proportion of residents with non recommended drugs (as defined by Swedish guidelines)	Non recommended hypnotics (+) RR 0.45 (0.35, 0.58) Non recommended anxiolytics (0) RR 0.96 (0.79, 1.16) Non recommended antidepressant (0) RR 0.67 (0.44, 1.03) Acceptable hypnotics (+) RR 1.46 (1.13, 1.89)
	Proportion of residents with acceptable drugs (as defined by Swedish guidelines)	Acceptable anxiolytics (0) RR 1.19 (0.97, 1.45) Acceptable antidepressant (-) RR 1.34 (1.07, 1.68)

Vu 2007 (Pseudo RCT)	Percentage healed	Follow up at 20 weeks Healed (0) - but baseline wound severity greater in intervention group Intervention 61.7%, control 52.5% p = 0.074

Involves pharmacists	Mean time to healing	Time to healing (mean days) (0) Intervention 82.0 (69.1-94.9), Control 101.1 (84.5-117.6), P = 0.095
	Total pain relief (Brief pain inventory)	Pain relief - BPI score = 0 (+) Intervention 38.6%, control 24.4% p = 0.017
	Costs	Mean treatment costs (+) Reduction in mean treatment costs of 357.7 Australian dollars when training costs included p = 0.004

### The nature of integrated working

There was a great deal of variation in how health care services and care homes worked together and the frequency of contact. For example, whilst some studies involved weekly multidisciplinary team meetings [43], monthly meetings were more common (Goodman, C et al: Can clinical benchmarking improve bowel care in care homes for older people? Final report submitted to the DoH Nursing Quality Research Initiative PRP, Centre for Research in Primary and Community Care, University of Hertfordshire, 2007)[30]. All the studies potentially increased care home staff access to health care professional's support and advice, with 15 out of 17 involving care home staff in multidisciplinary interventions or joint working. Care home staff were involved in multidisciplinary meetings and in some studies their opinions were sought [40], but they were led by health care professionals, with health care orientated and defined goals. Staff training was an integral part of all studies bar three; only a few studies consulted with care home staff on their perceived training needs [29,33]. The range of training input varied from as little as three hours [31] to seven seminars [37] or continuous training and support [43,44].

The level of integration for all studies and the degree of support and training provided by NHS staff for care home is reported in Table 8. The majority of studies showed micro integration at the clinical level involving close collaboration between care home staff and health care professionals to achieve specific outcomes (12 out of the 17) e.g. wound care techniques and wound healing. The remaining five studies were integrated at the clinical level but also showed greater complexity of integration in terms of funding and organisation or strategy, one at the meso level [42] and four at the macro level [31,41,43,44]. In service delivery, four studies used dedicated multidisciplinary teams to support staff and residents in care homes [42], three of which achieved their remit of avoiding unnecessary hospitalisation [41,43,44]. Two UK studies also had health service funded beds within care homes, one for use by a specialist health care nursing team [41] the other to provide end of life care [31]. A distinguishing feature of four out of the five studies classified at higher levels of integration was that care home staff received support and or training which was ongoing, as opposed to being offered at discrete time periods during the intervention. For example, nursing home staff were facilitated to recognise and manage acute conditions [43], to improve residents' overall care [44].

**Table 8:** Level of integration, care home staff support and training

Study	Model	1. Care staff involved in team meetings/joint working	2. Level of care home staff support	3. Training for care home staff	Training details	Level and features of integration
*Llewellyn-Jones, 1999*	Multidisciplinary case conferences	√	Duration of intervention only - no information on length	√	Duration of intervention only - no information on length	*Micro* Close collaboration between health care professionals and care home staff

*King, 2001*	Multidisciplinary consultation & collaboration	√ Senior nursing staff only	Duration of intervention only - 8 months	×	×	*Micro* Close collaboration between health care professionals and care home staff

*Opie, 2002*	Multidisciplinary consultation & collaboration	×	Duration of intervention Only - 4 weeks	×	×	*Micro* Close collaboration between health care professionals and care home staff

*Schmidt, 1998*	Multidisciplinary team meetings	√	Duration of intervention only 1 year	×	×	*Micro* Close collaboration between health care professionals and care home staff

*Vu, 2007*	Multidisciplinary consultation & collaboration	√	Duration of intervention only1 year	√	Training wound management. No details	*Micro* Close collaboration between health care professionals and care home staff

*Crotty, 2004*	Multidisciplinary case conferences	√	Duration of intervention only 1 year	√	Half day workshop on managing challenging behaviours	*Micro* Close collaboration between health care professionals and care home staff

*Joseph, 1998*	Multidisciplinary care	√	Ongoing weekly meetings to discuss deaths, hospitalisations and complications	√	6 hours of seminars every year. Ongoing training and feedback in the management of acute conditions	*Macro* Nurse practitioners employed to provide additional primary care Managed care Hospital avoidance

*Kane, 2004*	Multidisciplinary care	No information	Ongoing support but no details	√	Ongoing no information on the amount. Focus on training care home staff to improve resident's care	*Macro* Nurse practitioners employed to provide additional primary care Managed care Hospital avoidance

*Goodman, 2007*	Multidisciplinary consultation & collaboration	√	Duration of intervention only approximately monthly over 6 months	√	Duration of intervention One training session for care home staff in one care home	*Micro* Close collaboration between health care professionals and care home staff

*Szczepura, 2008*	Multidisciplinary care	√	Ongoing over 2 years	√	Ongoing over 2 years	*Macro* Dedicated nursing and physiotherapy In-reach team Dedicated care home beds Hospital avoidance Joint NHS - local authority initiative.

*Proctor, 1998*	Multidisciplinary Training - high level of staff involvement	√	Duration of intervention 6 months, weekly visits by specialist nurse	√	Duration of intervention - 7 one hour seminars by multidisciplinary team on topics chosen by care staff	*Micro* Close collaboration between health care professionals and care home staff

*Knight, 2007*	Collaborative working using integrated care pathways	√	Duration of intervention only 3 years	√	Duration of intervention only	*Micro* Close collaboration between health care professionals and care home staff

*Mathews, 2006*	Collaborative working using integrated care pathways	√	Duration of intervention only No information	√	Duration of intervention 3 hours on palliative care	*Macro* Close collaboration between health care professionals and care home staff Care pathways NHS funded bed

*Doherty, 2008*	Care home support team	√	Ongoing 1 year	√	Ongoing No details	*Meso* Dedicated care home support team established by NHS

*Hasson, 2008*	Link nurses in care homes	√	Duration of intervention, monthly meetings over 3 years	√	Duration of intervention only-nine 3 hour training sessions	*Micro* Close collaboration between health care professionals and care home staff

*Avis, 1999*	District nurses supporting care home staff	√	Duration of intervention only 2.5 years	√	Duration of intervention Only. At least 6 training sessions no details on length	Micro Close collaboration between health care professionals and care home staff

*Hockley, 2005*	Champions identified in care homes	√	Duration of intervention Only 1 year. Regular clinical support no information on frequency	√	Duration of intervention - Monthly collaborative learning and monthly action learning sets	*Micro* Close collaboration between health care professionals and care home staff

A number of cross cutting themes that influenced the achievement of integrated working were identified (See Tables 9 and 10). These included, care home access to services and the different working cultures of care home staff and health care professionals that acted as barriers and facilitators. Care home staff identified a lack of support from health care professionals and a failure to recognise their knowledge and skills [29,33,42]. There were negative perceptions on both sides with care home staff feeling that health care professionals were sometimes acting in a 'policing' rather than an advisory capacity [29,42] and health care professionals perceiving care home staff as lacking in knowledge and expertise, and unwilling to change their practice [30].

**Table 9:** Barriers to integrated working

	Barriers to integrated working
1.	Difficulty of NHS staff gaining the trust of care homes and NHS cynicism of care home expertise

2.	Lack of access to NHS services

3.	High staff turnover and lack of access to training

4.	Lack of staff knowledge and confidence

5.	Care homes were professionally isolated

6.	Lack of teamwork in care homes

**Table 10:** Facilitators to integrated working

	Facilitators to integrated working
1.	Care homes valued NHS input and training

2.	'Bottom up' approach to train staff so that all levels of staff are involved

3.	Health care professionals acting as a advocate for care homes in relation to care

4.	Health care professionals acting as facilitators for sharing good practice and enabling care home staff to network

5.	Health care professionals promoting better access to services for the care home

6.	Care home managers supporting staff access to training for example, through establishing learning contracts.

Whilst input and training from health care staff was valued, for care home staff to access it, dedicated time and finance from care home managers was necessary. Holding sessions within the care home and setting up a learning contract with the staff could facilitate training [32]. Examples of positive interactions included one care home support team described as acting as a link to 'the outside world' by the care home, and supporting clinical decision making across the multi disciplinary team [42]. Difficulty in maintaining levels of staff skills and knowledge were exacerbated by the high staff turnover experienced by care homes [29,32,33]. However, one study found a higher rate of staff turnover amongst the health care professionals involved in the intervention than the senior staff in the care homes (Goodman, C et al: Can clinical benchmarking improve bowel care in care homes for older people? Final report submitted to the DoH Nursing Quality Research Initiative PRP, Centre for Research in Primary and Community Care, University of Hertfordshire, 2007). Consistency of care home managers was identified as an important factor in building collaborative working with health care professionals [32].

## Discussion

We found 17 studies, eight of which were controlled evaluations. Although some of the studies reported positive outcomes most interventions had mixed or no effects when compared with the control group. There was insufficient information available to evaluate the cost of integrated working between care homes and primary health care professionals. Some of the qualitative studies suggested that integrated working had the potential to improve the quality of life for older people in care homes through increased support for care home staff and increased access to health care services. A small number of studies which were integrated at the macro or meso level, involved care homes that were supported by dedicated health service teams and health service funded beds or managed care, showed more positive outcomes such as avoidance of hospitalisation. They also differed from the micro integrated studies in their capacity to give ongoing support and training for care home staff, which had the potential to address one of the main identified barriers to integrated working and ultimately improve resident's care. This indicates that for integrated working to be successful, formal structures may need to be in place for health service delivery and organisation of care for care homes.

Despite the lack of evidence on effectiveness, studies consistently demonstrated key issues that supported or militated against integrated working. These findings are significant for future research and the development of interventions that rely on integrated working between health care services and care home staff. Barriers to integrated working included a failure to acknowledge the expertise of care home staff, their lack of access to health care services, as well as high care home staff turnover and limited availability of training. Facilitators to integrated working were the care home manager's support for the intervention, protected time and the inclusion of all levels of care home staff for training and support by health care professionals.

A common feature of the interventions was the use of multidisciplinary teams to improve one or more aspect of older people's health care. However, all the studies were led and conducted by health care professionals. There was no evidence of care home staff being involved in the definition or focus of the studies and some evidence that care home staff felt that their knowledge and views were not valued. Seven studies employed external project staff in some capacity, which implies that integrated working may require some external facilitation.

Three studies used integrated care pathways as a means of improving the quality of end of life care for older people resident in care homes. Care pathways may increase integrated working for the individual older people who have them, but this will not necessarily extend to the care home residents as a whole. The use of a shared assessment and care framework and documentation itself can become a useful source of continuity in an environment where there is high staff turnover and shift working in both sectors (Goodman, C et al: Can clinical benchmarking improve bowel care in care homes for older people? Final report submitted to the DoH Nursing Quality Research Initiative PRP, Centre for Research in Primary and Community Care, University of Hertfordshire, 2007).

### Limitations of existing evidence

Given the limited number of studies in the review, their heterogeneity, poor quality, small size, and low level of detail, the scope for discussion of integrated working between care homes and primary health care professionals is limited and firm conclusions cannot be reached. Only five studies were conducted in residential care homes which reinforced previous findings that the majority of research is carried out in nursing homes, even though this is not where most older people in long term care live [14]. The absence of older people's views and resident centred outcomes from the studies was notable.

Moreover the majority of studies were only integrated at the micro level that is, close collaboration between care home staff and professionals, so little information was available on the impact of integration at meso and macro levels. There was wide variation amongst the studies in terms of their level of care home staff support and training, and the involvement of older people. Care home staff training and support ranged between those studies where it was ongoing and those where it was provided only on one occasion. Where there was support and training of care home staff it was not clear if the ultimate aim was to train staff to a level of expertise so that health services could withdraw.

### Implications for research

There is a need for more research that addresses how integrated working can best be achieved and that evaluates the effect of integrated working on the health and wellbeing of older people, service use and cost. Research with care homes should reflect the context and constraints of working across public and independent services, and involve care homes in the planning and design of interventions. Moreover as this population is known to have multiple co-morbidities that are often compounded by cognitive impairment there is a need for more studies to look at improving the quality of care for the care home population as a whole. Future evaluations should be large enough to detect a difference and outcomes need to be meaningful to care home staff and residents.

### Strengths and limitations of the review

We used systematic and rigorous methods to synthesise the current evidence on integrated working between care homes and health care services and highlight areas for further research. There are, however, a number of methodological issues that could have a bearing on the validity of the results. Owing to a lack of evidence in this area we included all studies types including uncontrolled studies. Only four of our included studies were randomised controlled trials. Whilst uncontrolled studies might be more likely to be biased these broad inclusion criteria enabled us to investigate integrated working more widely and identify barriers and facilitators.

Although the studies reviewed were judged to have involved integrated working, it was not their main focus; only two studies referred to partnership working between care homes and health care services (Goodman, C et al: Can clinical benchmarking improve bowel care in care homes for older people? Final report submitted to the DoH Nursing Quality Research Initiative PRP, Centre for Research in Primary and Community Care, University of Hertfordshire, 2007.)[44]. The information on integrated working was based on how the intervention was described, who was involved and at what level. It is possible that how this was reported in the studies reviewed did not capture the extent of the integration achieved.

## Conclusions

Integrated working aims to ensure continuity of care, reduce duplication and fragmentation of services and places the patient as the focus for service delivery. This review identified a limited number of studies where the intervention supported integrated working between care homes and primary health care professionals. The narrow focus and single issue orientation of the majority of the studies did not engage with the needs of care home population or the context and organisation of their care. Outcome measures reflected the priorities of health care professionals rather than residents and care home staff. In view of the growing demand for residential and nursing home care together with funding constraints, more effective working between the NHS and care home providers is essential. There is an urgent need to develop and test interventions that promote integrated working and address the persistent divide between health services and independent providers.

## Competing interests

All authors declare: no support from any organisation for the submitted work; no financial relationships with any organisations that might have an interest in the submitted work in the previous three years; no other relationships or activities that could appear to have influenced the submitted work.

## Authors' contributions

CG, FB, SI, KF designed the protocol, SD, FB, CG, CV, KF screened literature, reviewed papers for inclusion, extracted data and wrote the paper. All authors interpreted data, critically reviewed the paper and have approved the final manuscript.

## Funding

This research was supported by the National Institute for Health Research Service Delivery and Organisation programme (project number 08/1809/231).

## Disclaimer

The views and opinions expressed therein are those of the authors and do not necessarily reflect those of the NIHR SDO programme or the Department of Health.

## Pre-publication history

The pre-publication history for this paper can be accessed here:

http://www.biomedcentral.com/1472-6963/11/320/prepub
